# Beyond Base Camp: Promise and Pitfalls of PI3K/mTOR Inhibition in Pediatric High- Grade Gliomas

**DOI:** 10.21203/rs.3.rs-6508597/v1

**Published:** 2025-05-05

**Authors:** Ryan J. Duchatel, Clara Savary, Zacary P. Germon, Madisen Riley, David S. Ziegler, Sabine Mueller, Evangeline Jackson, Matthew D. Dun

**Affiliations:** University of Newcastle; University of Newcastle; University of Newcastle; University of Newcastle; University of New South Wales; University of California San Francisco; University of Newcastle; University of Newcastle

**Keywords:** PI3K, Brain cancer, Diffuse Midline Glioma, Clinical Trials, High Grade Glioma

## Abstract

**Background:**

High-grade gliomas (HGGs), including diffuse midline glioma (DMG), represent the most aggressive and deadly pediatric brain cancers. Despite recent advances in understanding their molecular underpinnings, these tumors remain universally fatal. A hallmark feature of pediatric HGGs is the frequent presence of mutations and amplifications in components of the phosphatidylinositol-4,5-bisphosphate 3-kinase (PI3K) signaling pathway. These alterations drive unchecked tumor growth, confer resistance to standard therapies, and contribute to the dismal survival outcomes observed in affected children.

**Main Body:**

While the PI3K/mTOR axis has been recognized as a critical dependency in DMG and other pediatric HGGs, clinical translation of pathway inhibitors has been limited by several major barriers. Most notably, the blood–brain barrier (BBB) restricts the delivery of conventional PI3K and mTOR inhibitors, many of which lack sufficient central nervous system (CNS) penetration. Furthermore, even when delivered to the tumor site, these agents often encounter rapid adaptive resistance through activation of compensatory pathways, reducing their therapeutic benefit. Treatment-related toxicities, including hyperglycemia, rash, and mucositis, further limit tolerability and patient adherence.

Emerging brain-penetrant PI3K/mTOR inhibitors represent a new generation of targeted therapies with the potential to overcome these pharmacological limitations. However, increasing drug exposure does not necessarily equate to improved outcomes, particularly when used in combination with immunotherapies or other targeted agents. Achieving optimal therapeutic efficacy while minimizing systemic toxicity remains a central challenge, requiring careful consideration of drug dosing, timing, and combination strategies tailored to each individual patient.

**Conclusion:**

This review explores the current landscape of PI3K/mTOR targeting in DMG, highlighting both the therapeutic promise and inherent challenges. We discuss known resistance mechanisms, the need for better CNS-optimized compounds, and the importance of individualized treatment strategies. Finally, we propose a roadmap for future research, emphasizing rational drug combinations, refined patient stratification, and the development of next-generation therapies aimed at improving outcomes for children with these devastating malignancies.

## 1. Background

### 1.1 Opportunities and Challenges in Pediatric High-Grade Gliomas

Diffuse midline glioma (DMG) is the most common and lethal pediatric high-grade glioma (HGG), with a universally fatal prognosis. Median overall survival ranges from just 9 to 15 months [1]. with children diagnosed with diffuse lesions in the pontine region of the brainstem, termed diffuse intrinsic pontine glioma (DIPG), typically succumbing within a year of diagnosis. Palliative radiotherapy (RT) remains the only standard of care outside clinical trials. Reirradiation is increasingly used in longer-term survivors but offers only a modest survival benefit (4.2–6.9 months) [2, 3] underscoring the urgent need for effective therapeutic strategies. Mutations and amplifications in signaling genes *PDGFRA, PIK3CA/PIK3R1, FGFR, EGFR and ACVR1* coupled with the loss of function of tumor suppressors genes *TP53* and *PTEN* combine with hallmark instigating H3-alterations (*H3–3A, H3C2/H3C3, EZHIP*) to drive a highly clonal disease, limiting the benefit of single agents [4, 5]. Pharmacological inhibition of overexpressed or constitutively activated signaling pathways remain a key therapeutic strategy under intensive investigation for all cancers. However, past clinical trials for DMG have failed to improve survival, underscoring the urgent need for more effective therapeutic options. One of the major challenges in treating DMG is the blood-brain barrier (BBB), which significantly restricts drug delivery [6]. HGGs originating in the midline, such as DMGs, exhibit even lower BBB permeability (‘leakiness’) than other HGGs, including glioblastomas [7], further complicating efforts to develop pharmacological agents capable of improving patient outcomes.

## 2. Main Text

### 2.1 PI3K/mTOR Signaling in DMG: A Key Driver and Therapeutic Opportunity

**Figure 1:**
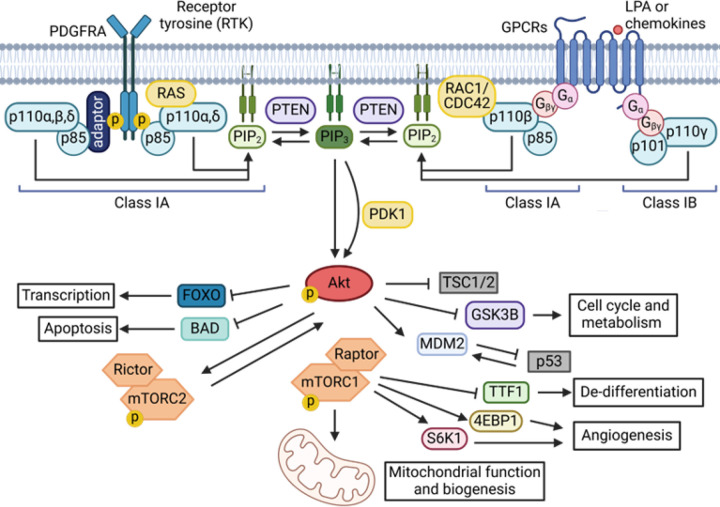
Signal transduction via PI3K/Akt/mTOR pathway promoting brain cancer growth and survival. The phosphatidylinositol 3-kinase (PI3K) pathway (light blue) is activated by receptor tyrosine kinases (RTKs) or G-protein–coupled receptors (GPCRs) upon binding of growth factors (e.g., PDGFRA, VEGF) or cytokines. This activation leads to the phosphorylation of phosphatidylinositol-4,5-bisphosphate (PIP_₂_, light green) to generate phosphatidylinositol-3,4,5-trisphosphate (PIP_₃_, dark green). PIP_₃_ recruits pleckstrin homology (PH) domain-containing proteins, including Akt (red), to the membrane, triggering downstream signaling cascades. Akt phosphorylates multiple targets to regulate key oncogenic processes such as transcription, cell cycle progression, metabolism, and apoptosis. Downstream, mTOR complex 1 (mTORC1, orange) promotes tumorigenesis by modulating autophagy, angiogenesis, and cellular de-differentiation. The tumor suppressor PTEN (purple) negatively regulates the pathway by dephosphorylating PIP_₃_ to PIP_₂_. Further regulation occurs through inhibition of tumor suppressor proteins including p53 and the TSC complex (both shown in grey), contributing to uncontrolled cell growth and survival in brain tumors. (Created in Biorender, adapted from [4, 14]).

One of the most important contributions to the malignant growth, metabolism, survival, and migration of HGGs is the phosphatidylinositol 3-kinase (PI3K) lipid kinase signaling pathway [8, 9]. The PI3K gene family is divided into three PI3K classes (I-III) based upon their different structures and substrates [10]. Functionally, the PI3K family is comprised of the catalytic subunit p110α (*PIK3CA*) and a regulatory subunit p85α (*PIK3R1* – including regulatory isoforms p55 and p50) [11]. Class I PI3Ks form a heterodimeric complex comprised of one of four catalytic isoforms - p110α (*PIK3CA*), p110β (*PIK3CB*), p110δ (*PIK3CD*), p110γ (*PIK3CG*) - and a regulatory isoform, which may be p85α, p55α, p50α (all encoded by *PIK3R1*), p85β (*PIK3R2*), p55γ (*PIK3R3*), p101 (*PIK3R5*), p84 or, p87 (*PIK3R6*) [11]. This class of PI3Ks is commonly found mutated in cancers, particularly p110α/*PIK3CA*. Upon activation, PI3K catalyzes the conversion of PIP2 to PIP3, a second messenger that recruits Akt to the membrane, where it becomes activated. Akt, in turn, activates numerous downstream targets that promote cell growth, survival, and metabolism (Figure 1).

Alterations in platelet-derived growth factor receptor alpha (*PDGFRA*) are also a frequent genomic event in DMG and strongly associated with the H3.3K27M mutations (30%) and drive downstream signaling through the PI3K/Akt/mTOR pathway [4, 5]. In addition, activating mutations are seen in *PIK3CA* (12%) across all H3 subtypes, *PIK3R1* (18%) in H3.3K27M and EZHIP subtypes, *TSC2* (2%) in EZHIP subtypes, *RPTOR* (1%) only in H3.3K27M subtypes, as well as *MTOR* (1%) in H3.3K27M (34), while loss of function of the tumor suppressor protein *PTEN*, the negative regulator of this pathway is observed in 19% of DMG cases [4, 5]. We recently analyzed the functional consequence of PI3K expression across 38 patient-derived DMG models encompassing all H3-subtypes [12], using a CRISPR/Cas9 loss of function screen approach. This demonstrated that the *PIK3CA* catalytic subunit of PI3K/Akt/mTOR signaling, as well as MTORC1, are genetic dependencies, independent of activating mutations in any of the PI3K subunits (*PIK3CA/PIK3R1/AKT/MTOR*). These findings were confirmed by knockout of *PTEN*, increasing growth [13]. Targeted deletion of *PIK3CA* using CRISPR-Cas9 expressed in a PI3K/mTOR wildtype H3.3K27M DMG cell line (SU-DIPG-XIII) completely ablated *in vitro* proliferation, highlighting the importance of PI3K signaling in DMG regardless the presence of activating mutations in PI3K-related genes [13].

This may be explained by several studies suggesting that phosphatidylinositol (PI) metabolism is upregulated in HGGs, contributing to constitutive activation of PI3K signaling and tumor progression. The PI3K/Akt/mTOR pathway, driven by PI-derived phosphoinositides, is frequently hyperactivated in HGGs due to mutations/alterations leading to elevated PIP3 levels and downstream Akt/mTOR activation [15, 16]. Metabolomic studies have demonstrated that these cancers exhibit altered lipid profiles, including increased levels of PIs, which may facilitate enhanced PI3K activity independent of activating mutation [13, 17]. Moreover, the higher the tumor grade, the more increased the PI3K pathway is, correlating with poor prognosis and therapy resistance [17]. Elevated PI levels may act as a catabolic driver of constitutive PI3K signaling, fueling glioma progression and metabolic adaptation, offering new targets for combination strategies.

Additional elegant studies have hypothesized that targeting PI3K/Akt/mTOR signaling is critical to improve the outcomes of cancer patients. More than 10 different clinical trials have evaluated the efficacy of compounds targeting PI3K/Akt or mTOR for the treatment of DMG (Table 1); unfortunately, none are yet to progress further than safety and early efficacy studies. Importantly, early stage clinical trial data in other cancers suggest that targeting co-activated pathways with combination inhibitors is more effective than monotherapeutic approaches, owing to the simultaneous inhibition of intrinsic and adaptive resistance mechanisms, further explored in Section 2.2.3 [18, 19].

### 2.1 The Role of PI3K Signaling in Neurodevelopment and Disease

PI3K signaling is essential for neurodevelopment, particularly during embryogenesis, where trophic factors such as Insulin-like Growth Factor-1 (IGF1) play a critical role [20]. PI3K is fundamental for normal brain size and function, with mutations in *PIK3CA* during embryonic development leading to severe overgrowth disorders known as PIK3CA-Related Overgrowth Spectrum (PROS) [21]. These disorders include bilateral dysplastic megalencephaly, hemi-megalencephaly, and focal cortical dysplasia, the latter being a major cause of intractable pediatric epilepsy [22]. Neuronal stem cells in the developing brainstem rely heavily on PI3K signaling, including PTEN, underscoring the pathway’s dual role: essential for survival, and capable of causing severe abnormalities when dysregulated.

Common *PIK3CA* hotspot mutations seen in children with brain overgrowth disorders are also found as obligate partners to H3K27M mutations in HGGs and DMG, including H1047R and E545K [23]. A conditional *Pik3ca* knock-in transgenic mouse model induced at embryonic day (E0.5), displayed progressive hydrocephalus, ventriculomegaly, and megalencephaly, leading to death before weaning. However, when the same mutations were activated postnatally, they did not cause PROS, suggesting that *PIK3CA*-driven overgrowth disorders arise only when mutations occur in embryonic neural progenitors [23]. Whether this same principle applies to *PIK3CA* mutations in HGGs remains unknown. Indeed, postnatal mice with activated *Pik3ca* transgenes responded to acute treatment with BKM-120 (buparlisib – discussed in Section 2.2.4), a brain-penetrant pan-PI3K inhibitor, showing significant anti-epileptic effects [24]. These findings suggest that PI3K inhibitors could offer a promising new approach for managing intractable pediatric epilepsy associated with PIK3CA-driven disorders.

### 2.2 Clinical Trials Targeting PI3K/mTOR Signaling in DMG

**Figure 2:**
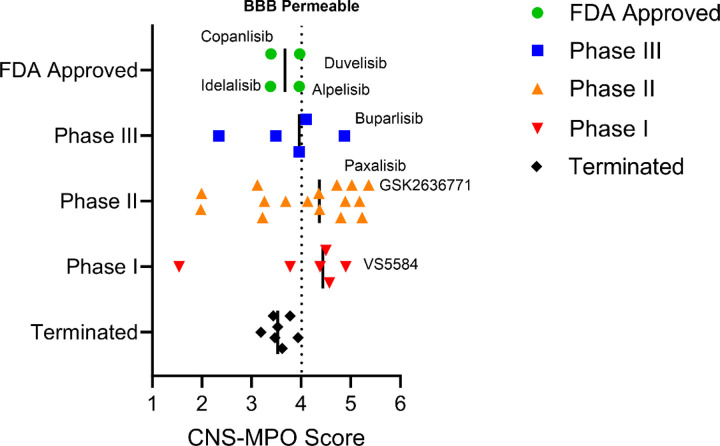
Clinical development of blood brain barrier-penetrant PI3K inhibitors. PI3K inhibitors currently in clinical development are shown, categorized by their clinical trial phase and assessed for predicted BBB penetration using the Central Nervous System Multiparameter Optimization (CNS-MPO) [41] scoring system. Compounds with CNS-MPO scores ≥4 are considered likely to penetrate the BBB, supporting their potential utility in treating brain tumors such as pediatric high grade gliomas (HGG) and diffuse midline glioma (DMG).

The *PIK3CA* gene is also mutated in 18% of breast cancers [31], 39% of endometrial [32] and 9% of non-small cell lung cancer [33], highlighting it as a key therapeutic focus for cancer treatment across almost all types [10]. Despite more than 40 inhibitors undergoing preclinical and clinical development, only a few have received FDA approval as anti-cancer therapies, including the pan-PI3K inhibitor copanlisib [34]. p110α (*PIK3CA*) inhibitor alpelisib [35, 36], p110γ/δ (*PIK3CY/PIK3CD*) inhibitor duvelisib [37], p110δ (*PIK3CD*) inhibitors idelalisib and umbralisib [37, 38]. However, for some (duvelisib, idelalisib, and umbralisib) accelerated approvals have been withdrawn [34]. Among mTOR inhibitors, mTOR class 1 inhibitors everolimus[39] and temsirolimus [40] have gained FDA approval. The limited number of approved inhibitors for clinical application can be attributed to toxicities associated with PI3K/mTOR inhibitors and their limited activity in the CNS, particularly in the context of brain tumors (Figure 2), and development of resistance. Many of these inhibitors are not approved for brain cancers, due to their low brain penetration as demonstrated by Central Nervous System – Multi parameter optimization (CNS-MPO) simulation results, which can predict CNS activity (Table 2). Therefore, the design and synthesis of new BBB-penetrant PI3K/mTOR inhibitors has been a focus for DMG treatment [13].

#### 2.2.1 Brain Penetration of PI3K/mTOR Inhibitors

To evaluate the potential of FDA-approved PI3K inhibitors for DMG, we applied the CNS-MPO scoring system. This system assesses six key physicochemical properties that influence a drug’s ability to penetrate the blood-brain barrier (BBB): calculated partition coefficient (ClogP), distribution coefficient (ClogD) at pH 7.4, molecular weight (MW), topological polar surface area (TPSA), number of hydrogen bond donors (HBDs), and the most basic center (pKa) [41].

Among FDA-approved PI3K inhibitors, copanlisib (CNS-MPO: 3.39), alpelisib (CNS-MPO: 3.96), duvelisib (CNS-MPO: 3.97), idelalisib (CNS-MPO: 3.98), and umbralisib (CNS-MPO: 1.99) suggest limited brain penetration (Table 2). Approved mTOR inhibitors scored even lower for both everolimus (CNS-MPO: 1.25) and temsirolimus (CNS-MPO: 1.00) suggesting that currently approved drugs have more active mechanisms of brain uptake, or are unlikely to be effective for brain tumors. However, there is an emerging trend of PI3K inhibitors in earlier phase clinical development, with increased brain penetration (Figure 2).

Newer PI3K/mTOR inhibitors demonstrate a more favorable CNS-MPO score: paxalisib (CNS-MPO: 4.44), buparlisib (CNS-MPO: 4.87), compound 7 (CNS-MPO: 4.72), and GCT-007 (CNS-MPO: 4.28) (Table 3). These findings suggest that while existing FDA-approved PI3K/mTOR inhibitors may have limited efficacy in HGG/DMG, next-generation compounds offer greater promise for BBB penetration and therapeutic potential.

#### 2.2.2 Hyperglycemia Linked to pan-PI3K inhibition

Hyperglycemia remains a significant challenge in the clinical application of PI3K inhibitors due to the PI3K/Akt pathway’s role in insulin-mediated glucose uptake [42]. Inhibiting this pathway disrupts glucose homeostasis, leading to circulating insulin and blood glucose. The effect is exacerbated using corticosteroids such as dexamethasone to manage peritumoral inflammation and hydrocephalus, further driving hyperglycemia and limiting the clinical benefit of PI3K inhibitors. Managing hyperglycemia and optimizing dosing strategies are critical for maximizing therapeutic efficacy while minimizing toxicity.

**Figure 3:**
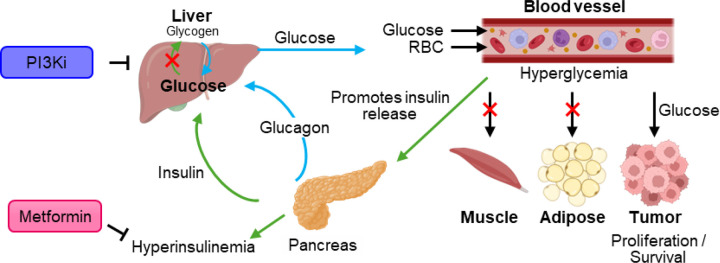
PI3K inhibition induces hyperglycemia and compensatory hyperinsulinemia. Inhibition of PI3K disrupts insulin signaling, impairing glucose uptake in insulin-sensitive tissues (e.g., muscle, adipose) and reducing hepatic glycogen synthesis. This leads to systemic hyperglycemia, which may paradoxically fuel tumor growth via enhanced glucose uptake in tumors overexpressing glucose transporters. Elevated glucose levels trigger compensatory insulin secretion, resulting in hyperinsulinemia. This metabolic feedback loop can be modulated by insulin-sensitizing agents such as metformin, which reduces hepatic gluconeogenesis and improves insulin sensitivity, potentially mitigating adverse effects of PI3K inhibition. (Created with Biorender, adapted from [13, 43, 44]).

PI3K/Akt signaling, particularly downregulating AKT2, facilitates insulin-driven glucose uptake in muscle, liver, and fat cells by promoting the glucose transporters translation to the plasma membrane [45]. Inhibiting, PI3K blocks this process, resulting in a dose-dependent increase in plasma fasting C-peptide and insulin, leading to systemic hyperglycemia [46]. This systemic insulin response reactivates PI3K/Akt signaling via insulin receptors, particularly in tumors with high level insulin receptor expression, thereby limiting the effectiveness of PI3K inhibitors [45] (Figure 3).

The power of combined glycemic control using oral anti-hyperglycemia medications and PI3K inhibitors was shown in the key study “SOLAR-1” of alpelisib in patients with *PIK3CA*-mutated hormone receptor positive breast cancer which led to improved progression-free survival and the FDA approval of alpelisib [47]. In this trial, 63.7% of alpelisib-treated patients experienced hyperglycemia of any grade with 36.6%, experiencing grade 3–4 hyperglycemia; with the discontinuation rate due to hyperglycemia of 6.3%. For patients experiencing any grade of hyperglycemia metformin was the first-line anti-diabetic agent used to manage alpelisib-induced hyperglycaemia, prescribed in 76% of affected patients either alone or in combination with other anti-diabetic medications.

#### 2.2.3 Resistance to PI3K/mTOR inhibition in DMG

Although PI3K inhibitors show promise in DMG, efficacy is often limited by compensatory pathway activation. Combining PI3K inhibition with agents targeting complementary oncogenic signals has improved tumor suppression, prolonged survival, and reduced invasion in preclinical models, offering a strategy to overcome resistance.

The combination of PI3K/mTOR inhibition (paxalisib) with MEK inhibition (mirdametinib) has shown synergistic efficacy in DMG patient-derived orthotopic xenograft (PDOX) models, where the combination significantly extended survival, whereas monotherapies did not [48]. Dual treatment more effectively inhibited both pathways in the brain and reduced MAPK signaling compared to mirdametinib alone, indicating disruption of compensatory resistance mechanisms. However, these studies did not assess hyperglycemia or evaluate co-treatment with metformin or optimized dosing strategies described in Section 2.2.2.

Despite the promise of PI3K inhibitors, efficacy in DMG is constrained by adaptive resistance mechanisms that preserve tumor proliferation and invasion. Protein kinase C (PKC) signaling, central to survival, migration, and progression, is a key bypass pathway. Our phosphoproteomic analyses reveal that PI3K inhibition with paxalisib increases PKC activity, where combining paxalisib with the PKC inhibitor enzastaurin showed strong efficacy in DMG models [13].

BDNF/NTRK2 signaling activates Ras/ERK and PI3K pathways, generating IP₃ and DAG to elevate intracellular Ca^2+^ and activate PKC, physiological mechanisms co-opted by DMG cells to enhance survival and invasion [49, 50]. We showed that paxalisib elevates Ca^2+^ levels and potentiates PKC activity, promoting migration and invasion. Co-treatment with the Ca^2+^ chelator BAPTA-AM abolished this effect, suppressing phosphorylation of pAKT, pPKC substrates, and pMARCKS, and significantly reducing invasion [13].

Hyperglycemia, a common consequence of PI3K inhibition, further activates PKC via increased DAG production, contributing to vascular dysfunction, angiogenesis, and tumor progression [51]. In HGG/DMG, corticosteroid use may amplify this effect. Our studies confirm that systemic hyperglycemia induced by PI3K inhibition at MTD correlates with enhanced PKC signaling and reduced efficacy. Moreover, PKC/MARCKS regulate adhesion, matrix remodeling, and apoptosis resistance, as shown by increased phosphorylation and invasiveness following PI3K/mTOR inhibition (paxalisib/rapamycin) or PKC activation (PMA), consistent with neuronal stimulation models [52–54].

The OPTIMISE trial (NCT06208657) evaluates paxalisib in combination with irinotecan and temozolomide (TMZ) for patients with PI3K/mTOR pathway alterations or progressive pHGGs, DMG, and DIPG (Table 1). Chemotherapy resistance in these tumors is frequently driven by robust DNA damage repair and an adaptive tumor microenvironment. PI3K/mTOR signaling supports both homologous recombination (HR) and non-homologous end joining (NHEJ), key pathways that repair genotoxic damage from agents like TMZ and irinotecan [55–58]. Paxalisib aims to inhibit these repair mechanisms, sensitizing tumor cells to chemotherapy and overcoming resistance.

DMG cells exhibit high metabolic plasticity, enabling adaptation to the fluctuating demands of rapid tumor growth and treatment-induced stress. As the PI3K pathway regulates key metabolic processes, this adaptability contributes to therapy resistance. Targeting mitochondrial function, specifically through activation of the mitochondrial protease ClpP, has emerged as a strategy to disrupt tumor metabolism and induce mitochondrial dysfunction in DMG.[59, 60] ONC201 (dordaviprone), which induces mitochondrial stress, has received Pediatric Rare Disease Designation and is currently in Phase 3 trials for DMG (NCT05580562) [61]. However, ONC201 also activates compensatory PI3K/Akt signaling, promoting metabolic adaptation and resistance [60, 61]. Understanding this metabolic ‘switch’ between glycolysis and oxidative phosphorylation is essential for overcoming resistance in current PI3K/mTOR-targeted trials, including PNOC022 (NCT05009992), where 20% of enrolled patients have survived beyond two years.

#### 2.2.4 Pan-PI3K Inhibitors in Clinical Development: Paxalisib and Buparlisib

Paxalisib (formerly GDC-0084), is a BBB penetrant pan-PI3K/Akt/mTOR inhibitor that inhibits all four isoforms of class I PI3K, tested in Phase I and II clinical trials for DMG (Table 1). The therapy was originally developed for the treatment of adult glioblastoma, which frequently harbors PI3K pathway alterations and is hyper-activated in 80% of cases due to deletions in *PTEN* [62][63].

A Phase I clinical trial for recurrent or progressive HGG (NCT01547546), reported disease stabilization in 40% of the 47 patients, but 11% of patients experience grade 3 adverse events (AE), including hyperglycemia and fatigue with mucositis identified as the primary dose-limiting toxicity (Table 1) [26]. In DMG/DIPG a Phase I dose-escalation study has been completed in the upfront setting, identifying a maximum tolerated dose (MTD) of 27 mg/m^2^/day (NCT03696355). Our lab discovered paxalisib as a potential therapy for DMG and recently published comprehensive mechanistic studies demonstrating dose optimization and the use of metformin to mitigate hyperglycemia, resulting in improved DMG model survival *in vivo* [13, 64].

Clinical trials testing paxalisib in DMG, both as monotherapy (NCT03696355) and in combination with ONC201 (NCT05009992), have reported PI3K inhibitor-related side effects, particularly mucositis, rash, colitis and hyperglycemia. Mucositis is now effectively managed with dexamethasone mouthwash (NCT05009992), but rash remains a challenge. The PI3K/Akt pathway plays a crucial role in insulin signaling, and concurrent corticosteroid therapy, commonly used to manage peritumoral inflammation and hydrocephalus in DMG, further exacerbates glucose dysregulation.

paxalisib-induced hyperglycemia in mice and found that treatment at the human-equivalent MTD (10 mg/kg/qd) induced systemic hyperglycemia, consistent with reports in glioblastoma models [65].

Dose modification, either half MTD daily or twice daily (13.5 mg/m^2^ human equivalent), ameliorated glucose disturbances. Pharmacodynamic analysis of DMG tissues from PDX mice confirmed robust inhibition of pThr308AKT (PI3K) and pSer473AKT (mTOR) at MTD, maintained with half MTD twice daily but once daily [13].

In efficacy studies, half MTD daily did not improve survival, while MTD extended survival by 10% daily, and half MTD twice daily by 17% compared to vehicle and MTD daily. Co-treatment with metformin (125 mg/kg/qd) further improved survival by 15% (half MTD daily) and 5% (half MTD twice daily), but not at MTD daily, suggesting high-dose PI3K inhibition overrides metformin’s protective effects [13]. These findings emphasize the need for dose optimization to maximize efficacy while minimizing metabolic toxicity in DMG.

Consistent with our findings, Noch et al., [42] reported in glioblastoma *in vivo* models, that paxalisib at the adult MTD (15 mg/kg/qd) extended survival, which was further enhanced by metformin (200 mg/kg/qd); combination therapy yielded the most pronounced reductions in p-AKT and p-S6 levels on histological analysis.

Buparlisib (BKM-120) is a BBB penetrable pan-PI3K inhibitor targeting all four class I PI3K isoforms (p110α, p110β, p110δ and p110γ) and is under clinical development for various brain cancers (Table 1). In glioblastoma, it demonstrated potent anti-migratory effects *in vitro* and slowed tumor progression in intracranial xenograft models [66]. However, clinical trials in PI3K-activated recurrent glioblastoma (NCT01339052, Table 1) showed limited benefit, with failure attributed to incomplete downstream PI3K pathway inhibition [29, 42]. Indeed Noch et al., using data from the Phase 2 study evaluating buparlisib in patients with recurrent GBM (NCT05183204) show that buparlisib activation of insulin signaling promoting hyperglycemia were independently associated with poor prognosis [29].

These challenges are not unique to HGG and DMG. In a clinical trial for brain-metastatic triple-negative breast cancer (NCT01629615), clinical benefit from PI3K inhibition was observed in only 12% of patients, with significant toxicities, including hyperglycemia, rash, and fatigue, similar to those seen with other pan-PI3K inhibitors [67]. Notably, these studies did not incorporate concurrent anti-glycemia medications.

Immune-related adverse events associated with agents like paxalisib and buparlisib may stem from their pan-PI3K activity. Inhibition of the p110δ isoform, primarily expressed in hematopoietic cells, reduces macrophage recruitment and preferentially suppresses regulatory T cells (Tregs) [68]. While potentially enhancing anti-tumor immunity, this effect has been linked to hepatotoxicity, colitis, pneumonia, and even intestinal perforation, limiting clinical utility by constraining dosing and drug exposure at the tumor site.

### 2.3 Novel PIK3CA and mTOR inhibitors: GCT-007 and Compound 7

GCT-007 (Global Cancer Technology) is a brain-penetrant, highly selective inhibitor of p110α the catalytic subunit of PI3Kα (PIK3CA), making it a promising candidate for HGGs. Unlike pan-PI3K inhibitors, GCT-007 targets the most mutated PI3K isoform in cancers, minimizing off-target effects while maintaining robust anti-tumor activity. Preclinical studies in glioblastoma have demonstrated that GCT-007 effectively inhibits tumor growth both *in vitro* and *in vivo* [69], supporting its therapeutic potential for other brain malignancies.

Currently, GCT-007 is under investigation for its efficacy in multiple cancers, including breast cancer and psoriasis, with ongoing research evaluating its utility in DMG within our laboratory. Given its upstream position in the PI3K/Akt/mTOR signaling cascade, GCT-007 offers an opportunity to broadly suppress downstream oncogenic signaling, potentially mitigating compensatory activation of parallel survival pathways. The ability to selectively target PIK3CA may also improve tolerability and reduce the metabolic toxicities commonly associated with pan-PI3K inhibition.

Compound 7 (Novartis) is a highly BBB-penetrable dual inhibitor of both mTOR complexes (mTORC1 and mTORC2) [70]. Among PI3K pathway inhibitors, BBB permeability remains a critical challenge with most mTOR inhibitors failing to effectively reach the tumor sites in the CNS (Table 2). Aside from Compound 7, PQR620 is the only other reported selective dual mTORC1/mTORC2 inhibitor with demonstrated BBB penetrance [71].

Beyond its potential application in cancer, Compound 7 has shown promising results in models of neurological disorders, highlighting its role in regulating mTOR-dependent neurodevelopmental and neurodegenerative processes [71]. Notably, in neuronal cell-based models, Compound 7 successfully inhibited mTOR signaling and extended the survival of murine models harboring neuron-specific loss of *Tsc1*, a negative regulator of mTOR [70].

While direct inhibition of mTOR presents a compelling strategy to block PI3K/Akt/mTOR signaling, it is important to consider whether targeting PIK3CA upstream may offer a more effective therapeutic approach. By inhibiting p110α at the top of the cascade, may prevent compensatory activation of alternative survival mechanisms downstream. Given that mTOR inhibition alone does not address PI3K-driven hyperglycemia, a key metabolic challenge in PI3K-targeted therapies, selective p110α inhibitors like GCT-007 may provide a more integrated approach to pathway suppression in DMG. Future studies should assess the comparative efficacy of p110α and dual mTORC1/2 inhibition *in vivo* to determine the most effective strategy for suppressing PI3K pathway activity while minimizing resistance.

### 2.4 PI3K/mTOR Inhibition Improves Immunotherapy Efficacy

**Figure 4:**
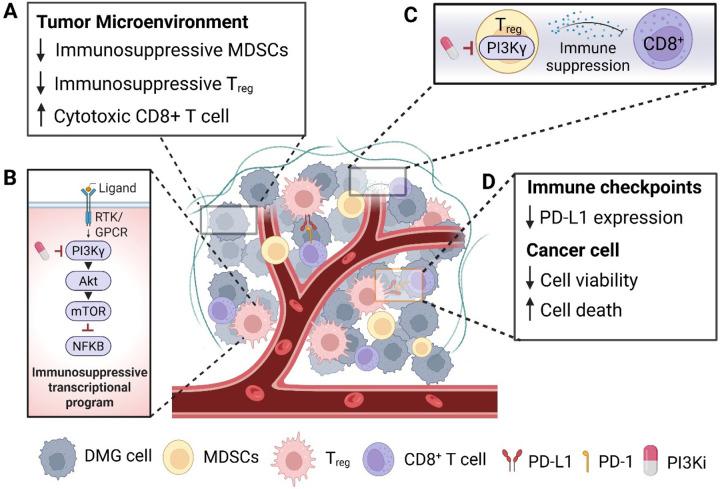
PI3K/Akt/mTOR inhibition enhances anti-tumor immune responses. **(A)** Regulatory T cells (Tregs) suppress effective anti-tumor immunity. Selective inhibition of PI3Kγ reduces Treg abundance, correlating with increased infiltration of CD8+ T cells expressing activation markers such as granzyme B [74, 76, 77]. **(B)** In myeloid-derived suppressor cells (MDSCs), PI3Kγ activation suppresses NF-κB signaling via Akt/mTOR, driving an immunosuppressive transcriptional program [74]. **(C)** Inactivation of PI3Kγ in MDSCs reverses this suppression, promoting an immunostimulatory phenotype that restores CD8+ T cell activity and cytotoxicity [76]. **(D)** PI3K/Akt/mTOR signaling also regulates expression of immune checkpoints such as anti-programmed death protein 1(anti-PD-1) in the tumor but also in tumor associated macrophages (TAMs) by epigenetic mechanisms [72]. (Created with Biorender).

Emerging evidence suggests that inhibition of the PI3K/mTOR pathway enhances the efficacy of immunotherapy through multiple mechanisms, including modulation of the tumor microenvironment, epigenetic reprogramming, and direct effects on immune cell function [72]. PI3K/mTOR signaling plays a key role in regulating the expression of immune checkpoints such as PD-L1, with inhibition shown to downregulate PD-L1 expression via epigenetic mechanisms including histone deacetylation and methylation changes (Figure 4) [73–75].

In preclinical and clinical studies, inhibition of PI3K (particularly PI3Kγ and PI3Kδ in myeloid and lymphoid cells, respectively) promotes the infiltration and activation of cytotoxic CD8+ T cells while reducing immunosuppressive regulatory T cells and myeloid-derived suppressor cells (MDSCs) (Figure 2) [74, 76, 77].

Furthermore, culturing chimeric antigen receptor (CAR) T cells in the presence of PI3K or mTOR inhibitors during *ex vivo* expansion can improve their metabolic fitness, persistence, and anti-tumor efficacy upon infusion. Agents such as idelalisib, rapamycin, and metformin, the latter activating TSC1/2 through AMPK and thereby inhibiting mTORC1,[78] have been shown to promote the development of memory-like T cells with greater *in vivo* persistence and cytotoxicity [79–81].

However, the timing and dosing of PI3K/mTOR inhibition are critical. Excessive inhibition, particularly systemic or sustained high-dose administration, can lead to immunosuppression by impairing T cell proliferation and survival, effectively negating the benefits of immunotherapy [13, 82]. This has been demonstrated in our own studies optimizing pharmacokinetics (PK) and pharmacodynamics (PD) of PI3K/mTOR-targeted therapies in DMG, where we observed a narrow therapeutic window. Thus, careful balance is required to enhance immune response without compromising immune cell viability [13].

Given the immunologically “cold” nature of pediatric HGGs and DMGs - characterized by low levels of T cells - rationally combining the immunostimulatory effects of PI3K/mTOR inhibitors with ICIs or CAR T cells may represent a critical avenue to overcome resistance and drive more durable anti-tumor responses. Future studies should focus on defining optimal schedules, dosing, and patient selection to maximize the synergistic potential of these multimodal strategies.

## 3. Conclusions

PI3K and mTOR represent clear genetic dependencies in pHGG, including DMGs. Recent advances in the development of brain-penetrant pan- and selective PI3K and mTOR inhibitors provide the field with potent new weapons against these historically lethal pediatric brain cancers. However, despite these promising tools, there remains substantial work to do if we are to meaningfully improve patient outcomes.

Here, we have outlined the advantages and limitations of both pan- and isoform-selective PI3K/mTOR inhibitors that can reach the brain. Selective inhibitors may reduce systemic toxicity and enhance tumor-targeted efficacy, whereas pan-inhibitors, while potentially more toxic, may yield superior responses when dosed optimally or combined with immunotherapies. Emerging studies support the potential of both approaches, but as a field, we must take greater care to interpret and apply these insights if we are to move forward effectively.

The highly adaptive nature of the DMG epigenome, driven by hallmark H3-alterations, necessitates rational combination strategies to overcome therapeutic resistance. Given the tumor’s capacity to activate compensatory survival pathways, single-agent PI3K inhibitors are unlikely to produce durable responses. Instead, integrating PI3K/mTOR inhibition with complementary therapies that target parallel or downstream mechanisms may hold greater promise for long-term control.

The path forward, moving PI3K/mTOR inhibitors from research and early clinical development and then toward regulatory approval for pHGGs, is complex. We must not only improve therapeutic outcomes but also ensure that this endeavor remains viable and attractive for researchers and industry partners. This means optimizing therapeutic windows, selecting synergistic drug combinations, managing toxicities, and aligning treatment with immunotherapy strategies. Equally important is identifying the patients most likely to respond.

We remain hopeful that, over the coming years, these challenges will be addressed. With sustained collaboration and innovation, we can climb beyond the preclinical *Base Camp* and begin the final ascent toward the summit of the *most lethal mountain* in pediatric oncology-DMG.

## Key Points

1. High-grade gliomas, including diffuse midline gliomas, show dependency on the PI3K/mTOR pathway, with frequent somatic alterations in associated genes also seen in patients.
2. The new brain penetrant PI3K/mTOR offer hope for improved precision guided treatment of DMG.
3. Children are not adults, clinical trials have overlooked key limitations of PI3K/mTOR inhibition in pediatric brain tumors, including poor brain penetration, toxicity, and resistance.
4. Next-generation PI3K inhibitors, combined with anti-glycemic therapies, may enhance efficacy in pediatric brain cancer.
